# Supplementary material for: Morphogenesis of extra-embryonic tissues directs the remodelling of the mouse embryo at implantation
Source: Nat Commun. 2019 Aug 7;10:3557. doi: 10.1038/s41467-019-11482-5 (PMC6686005; doi:10.1038/s41467-019-11482-5)
Supplement: Supplementary file 1 — Supplementary information [file 41467_2019_11482_MOESM1_ESM.pdf]

Supplementary Figures

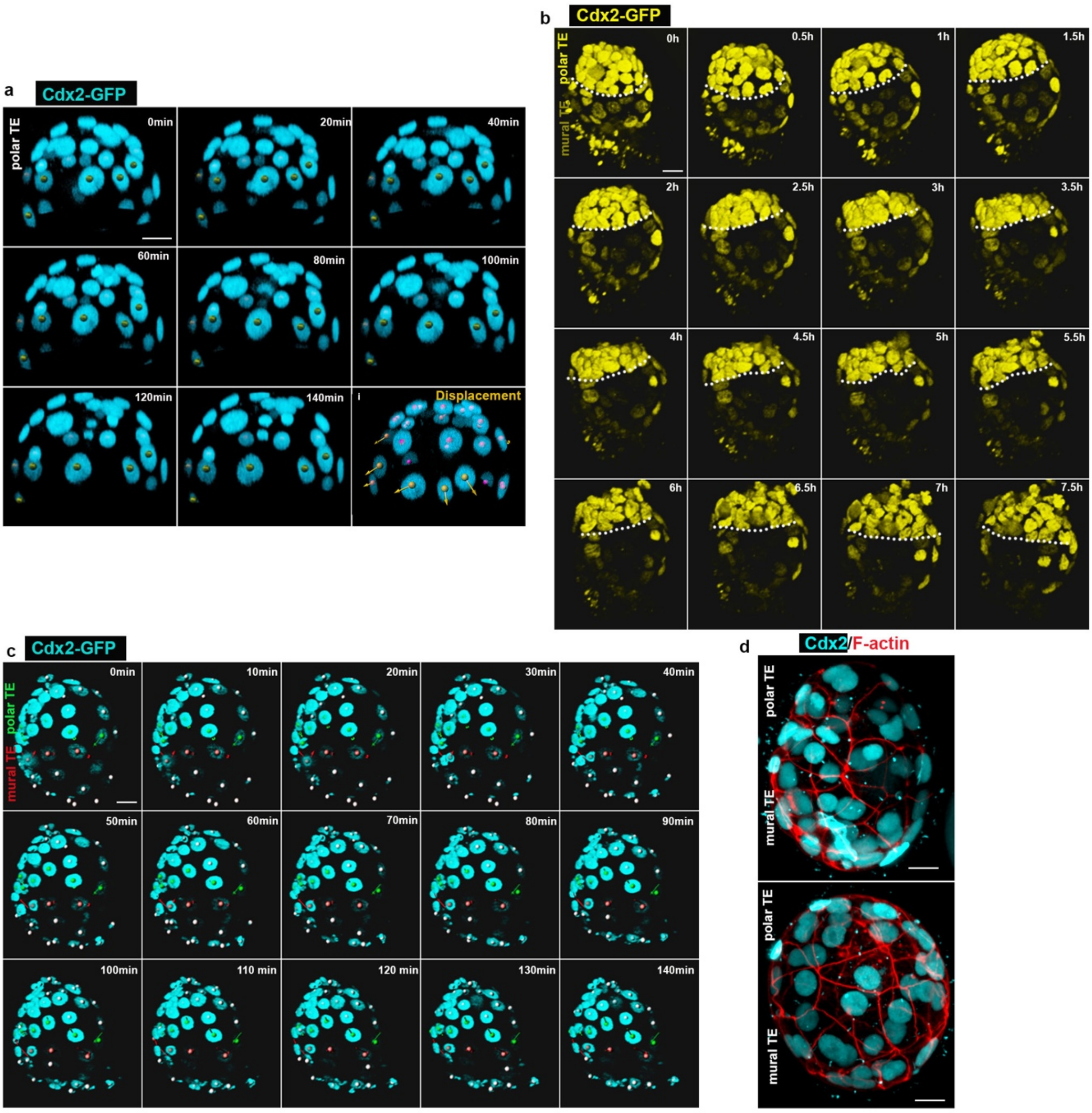

Supplementary Figure 1. Trophectoderm cell flow during preimplantation and implantation development (a) Stills from a time lapse movie of a Cdx2-GFP E3.5 blastocyst showing flow of cells from polar to mural TE. Yellow dots: tracking of boundary polar-TE cells. Bottom right panel: Displacement map of border polar-TE cells. (b) Stills from a time lapse movie of Cdx2-GFP E4.5 implanting blastocyst showing stop of cell flow from polar to mural TE upon implantation. CDX2 shows gradient expression with higher levels in polar TE. Dotted white line: polar/mural TE border. (c) Stills from a time lapse movie of Cdx2-GFP E4.5 implanting blastocyst combined with the displacement map for border polar and mural TE cells. (green: polar TE; red: mural TE) showing stop of cell flow from polar to mural TE upon implantation. (d) Cdx2 expression in pre-implantation (E3.5) blastocysts. Representative of 20 embryos. Scale bars= 20um.

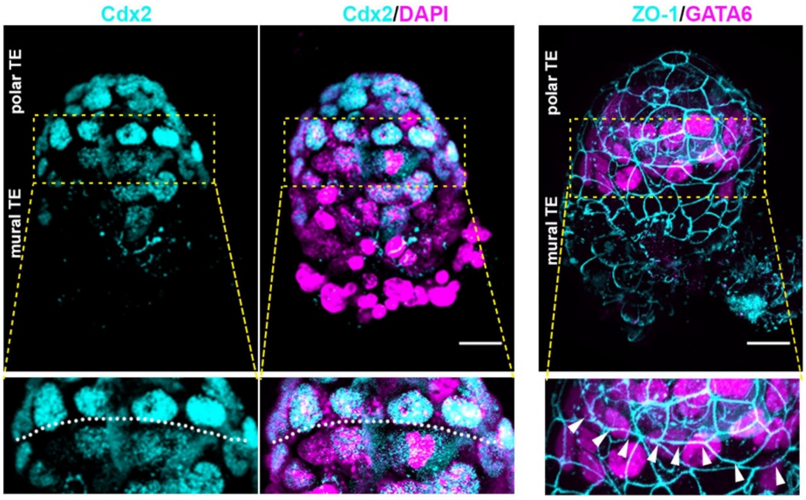

Supplementary Figure 2. Multipotency gradient within the trophectoderm. Cdx2 expression and polar/mural TE tissue boundary formation in implanted E4.75 blastocyst. White line marks the border between the polar and mural TE. Arrowheads indicate the linearity of cell junction at the polar/mural TE tissue boundary. Scale bars= 20um.

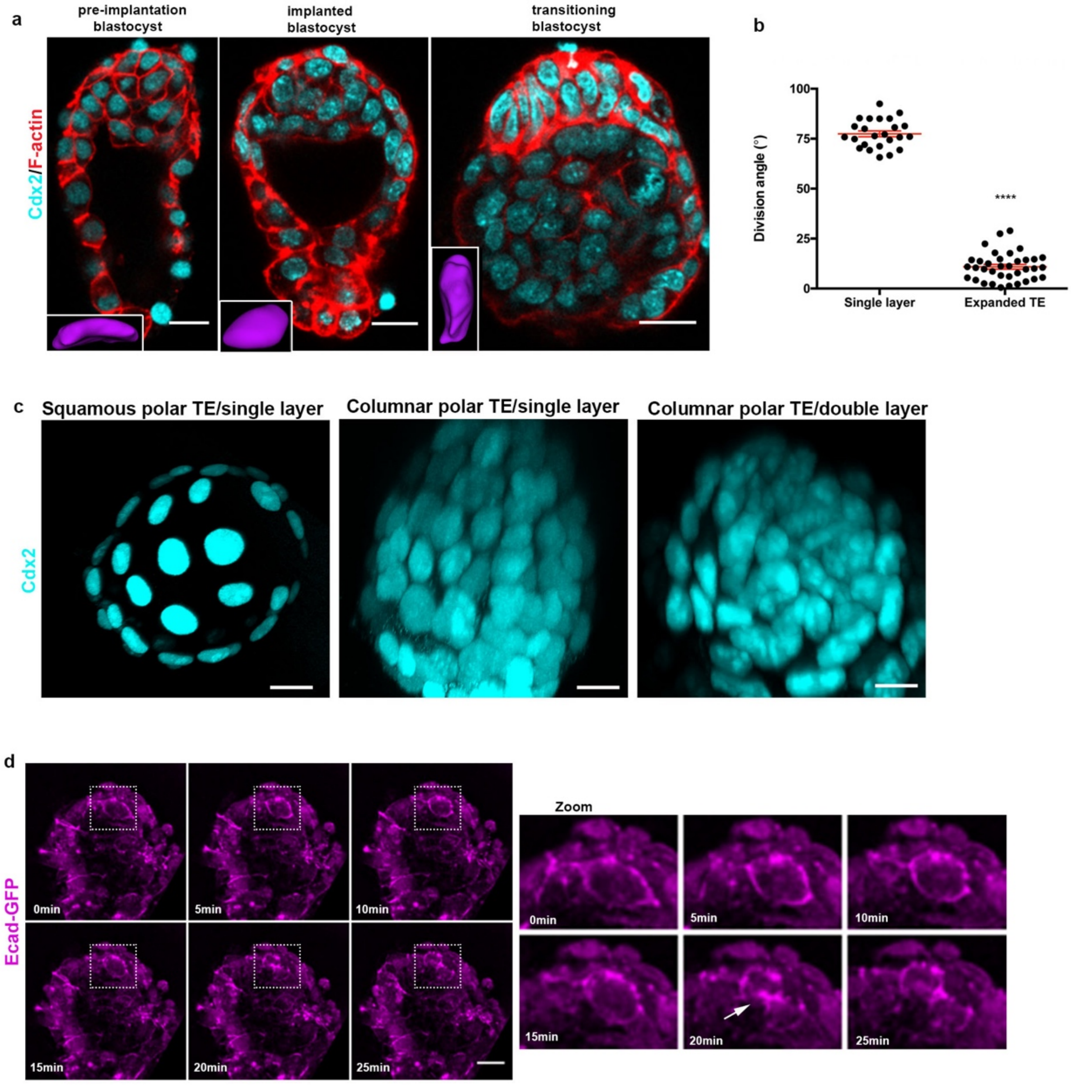

Supplementary Figure 3. Trophectoderm cell behaviour during tissue expansion (a) Examples of embryos at different peri-implantation stages used for analysis of polar TE cell shape changes. Representative of 30 embryos per stage. (b) Quantification of cell division orientation in blastocysts before and during polar TE expansion. Data are visualised as rose diagram of frequency distribution in Figure 2j. Two-sided unpaired student's t-test; ****$P<0.0001$; mean±SEM. Blastocyst before polar TE expansion: n= 24; Blastocyst during polar TE expansion: n= 36. Scale bars= 20um. c) 3D views of polar TE at different developmental stages. Cell density increases concomitantly with polar TE cell shape changes. (d) Stills from a time lapse movie of Ecad-GFP implanting blastocyst. Zoom indicates a dividing polar TE cell. Division occurs parallel with the epithelial layer. Source data are provided as a Source Data file. Scale bars= 20um.

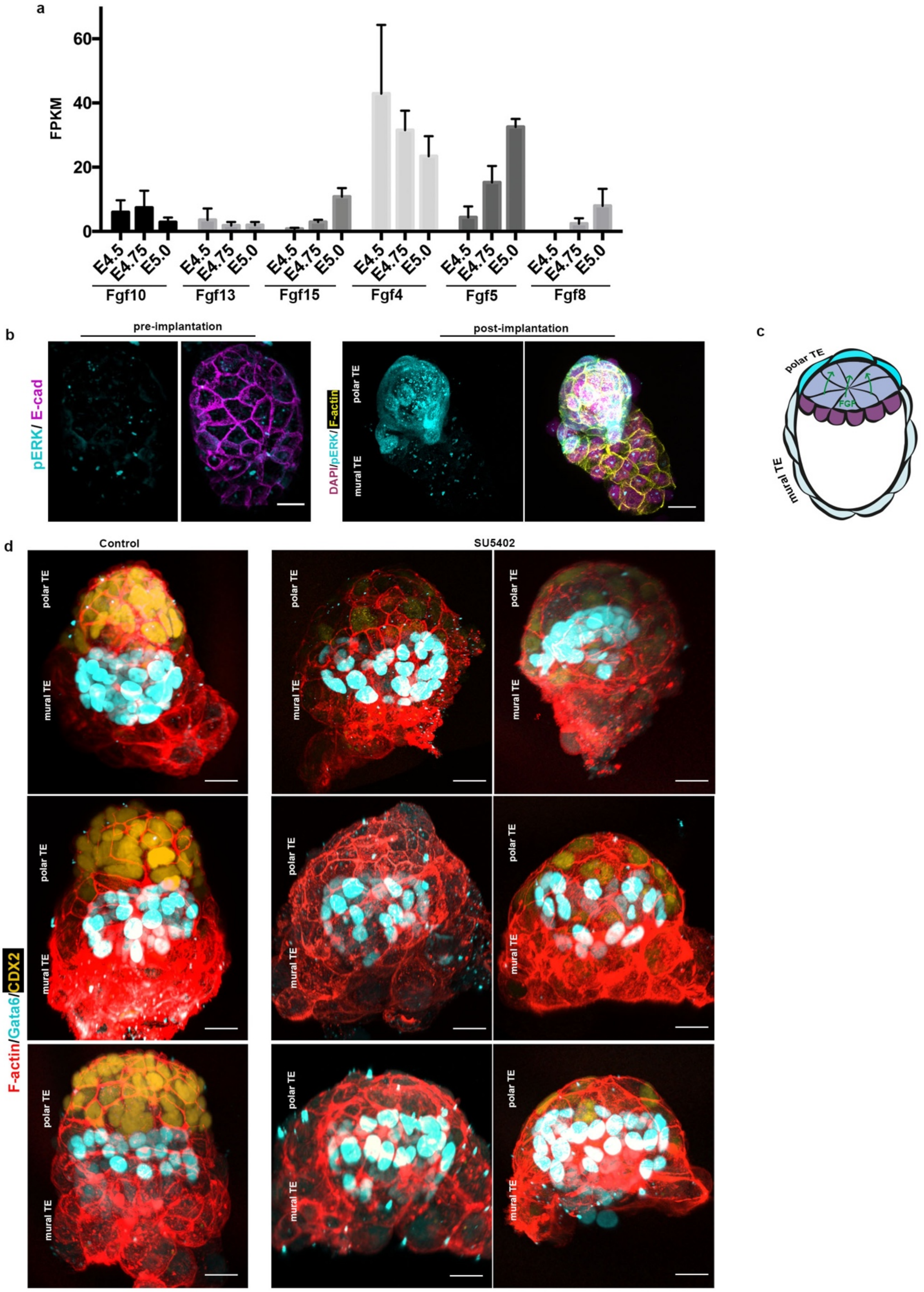
a
FPKM
0
20
40
60
E4.5
E4.75
E5.0
Fgf10
Fgf13
Fgf15
Fgf4
Fgf5
Fgf8
b
pre-implantation
post-implantation
pERK/ E-cad
DAPI/pERK/F-actin
polar TE
mural TE
c
polar TE
mural TE
FGF
d
Control
SU5402
F-actin/Gata6/CDX2
polar TE
mural TE

Supplementary Figure 4. FGF signalling is necessary for trophectoderm multipotency maintenance. (a) Expression of FGF ligands from RNA-seq database during peri-implantation development[1]. (b) Pre-implantation (E4.5) and post-implantation (E4.75) blastocyst (E4.75) stained for phosphorylated ERK (pERK) to reveal Fgf signalling activity. Fgf activity is detected only in the trophectoderm (TE) of post-implantation blastocysts. pERK is restricted only at the polar region of the TE. Representative examples of 10 embryos. (b) Schematic displaying the action of Fgf signalling in the implanting blastocyst. Cyan: increase pERK levels in the polar TE. (d) Representative examples of control (3 embryos) and SU5402 treated(20h) blastocyst (6 embryos) (Figure 2b-c) stained for the trophectoderm marker (Cdx2) and primitive endoderm marker (Gata6). Treatment of implanting blastocyst with Fgfr inhibitor doesn't affect the primitive endoderm lineage. Scale bars= 20um.

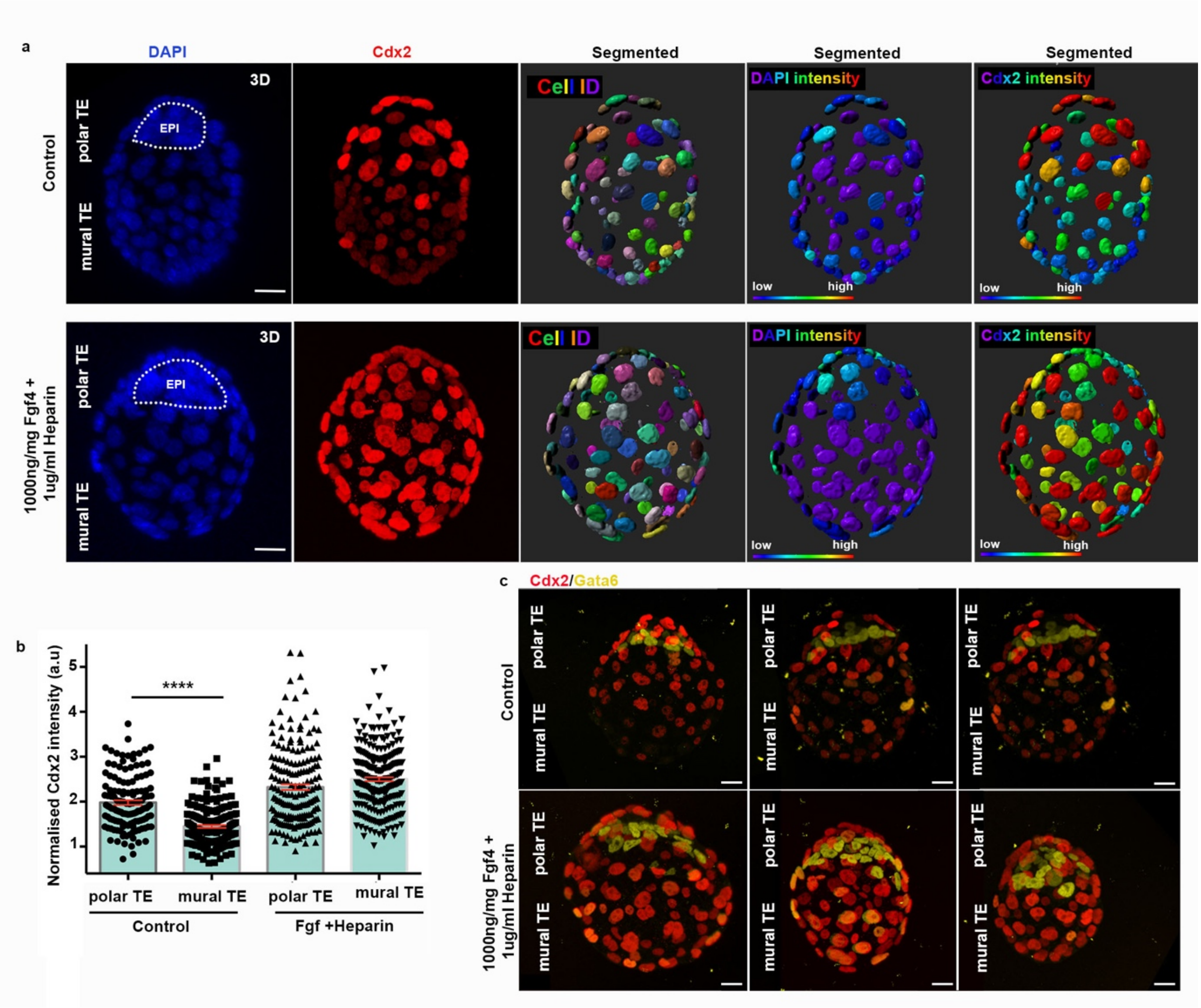

Supplementary Figure 5. FGF signalling is sufficient for trophectoderm multipotency maintenance. (a) Representative examples of control and Fgf treated embryos. Embryos were recovered at E3.5 cultured for 24h in KSOM and then cultured for another 24h in IVC medium in the presence or the absence of 1000ng/ml Fgf4 + 1ug/ml Heparin. Cdx2 levels are higher in the polar TE only in control embryos. Mural TE differentiation is defective in treated blastocysts. This is indicated by the absence of Cdx2 gradient from treated blastocysts. EPI: epiblast. (b) Quantification of Cdx2 intensity levels within polar and mural TE in control and Fgf4 treated embryos. χ^2 test ; ****P<0.0001, mean±SEM. Control embryos: n= 135 polar TE and 200 mural TE cells. Treated embryos: n=185 polar TE and 248 mural TE cells. (c) Representative examples of control and FGF treated embryos. Embryos were recovered at E3.5 cultured for 24h in KSOM and then cultured for another 24h in IVC medium in the presence or the absence of 1000ng/ml Fgf4 + 1ug/ml Heparin. Primitive endoderm specification (Gata6) is not affected by Fgf treatment at this developmental period. Source data are provided as a Source Data file. Scale bars=20um.

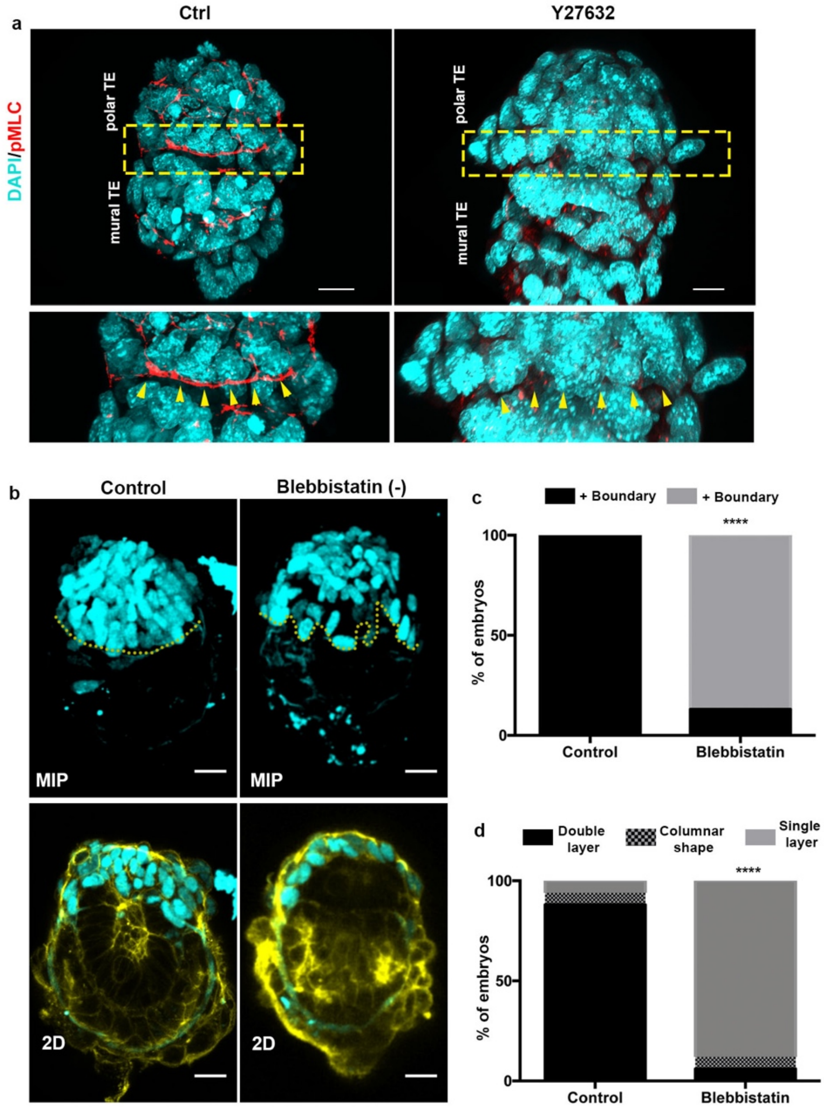

Supplementary Figure 6. Trophectoderm tissue boundary formation depends on actomyosin contractility (a) Representative example of a control and a Rock inhibitor (Y27632 treatment for 20h) treated embryos. Phosphorylated myosin at the polar/mural TE boundary interface (arrowheads in zoom image) is lost upon ROCK inhibition. (b) Representative examples of control, and blebbistatin (-) treated embryos. Embryos were cultured for 20h and analysed for the presence of polar/mural TE boundary (yellow dotted line in bottom panel). Polar/mural tissue boundary formation is defective in the blebbistatin treated embryos(MIP: Maximum Intensity Profile images upper panels), and this results in defective polar TE expansion(bottom panels). (c) Quantification of polar/mural TE boundary formation efficiency in control, and blebbistatin(-) treated blastocysts. χ^2 test ; ****P<0.0001, mean±SEM n= 16 control, 16 and 16 blebbistatin(-) treated embryos. (d) Quantification polar TE expansion efficiency in control and blebbistatin(-) treated blastocysts. χ^2 test ; ****P<0.0001. n= 16 control 16 blebbistatin (-) treated embryos. Scale bars=20um

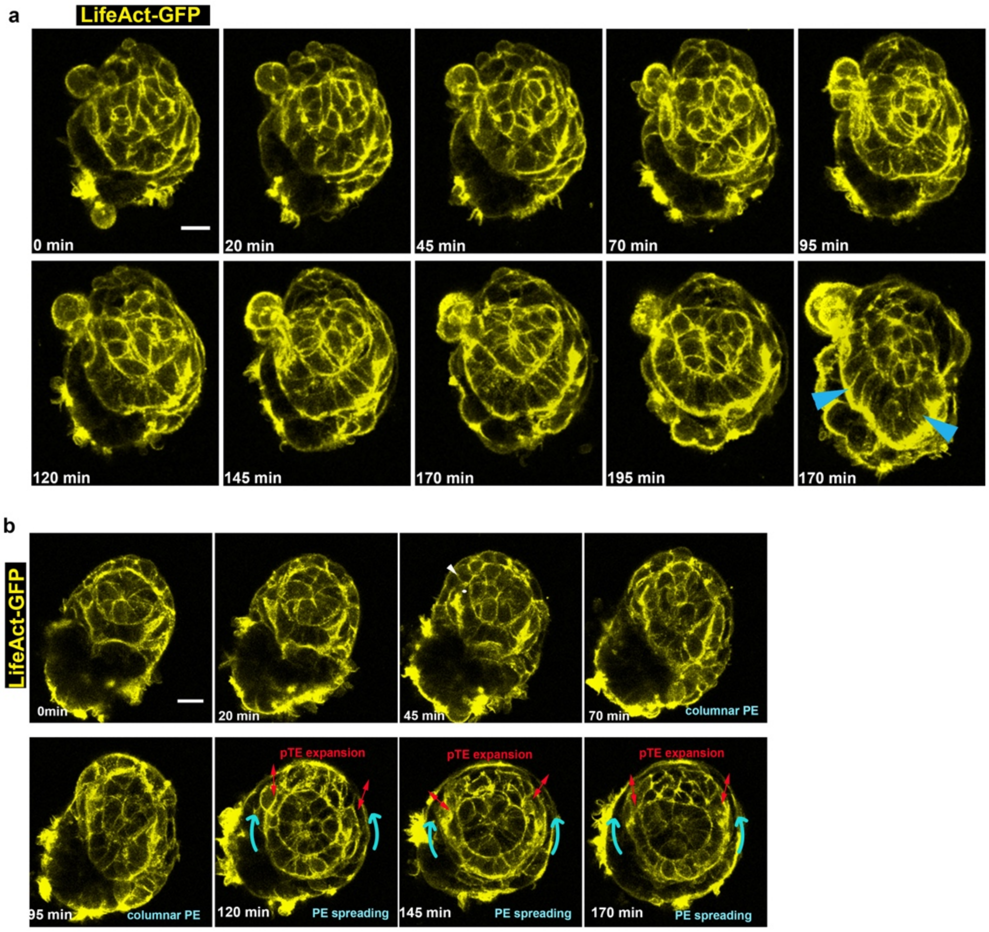

Supplementary Figure 7. Cell shape changes within the primitive endoderm (a) Stills from a time lapse movie of Lifeact-GFP E4.5 implanting blastocyst. Cyan arrowheads indicate the columnar shape acquired by primitive endoderm cells. (b) Stills from a time lapse movie of Lifeact-GFP E4.5 implanting blastocyst. PE acquires a columnar morphology at implantation stages and this is followed by tissue spreading through cell-shape changes upon polar-TE expansion. Red double-headed arrows: polar TE expansion. Cyan arrows: PE spreading. Scale bars= 20um.

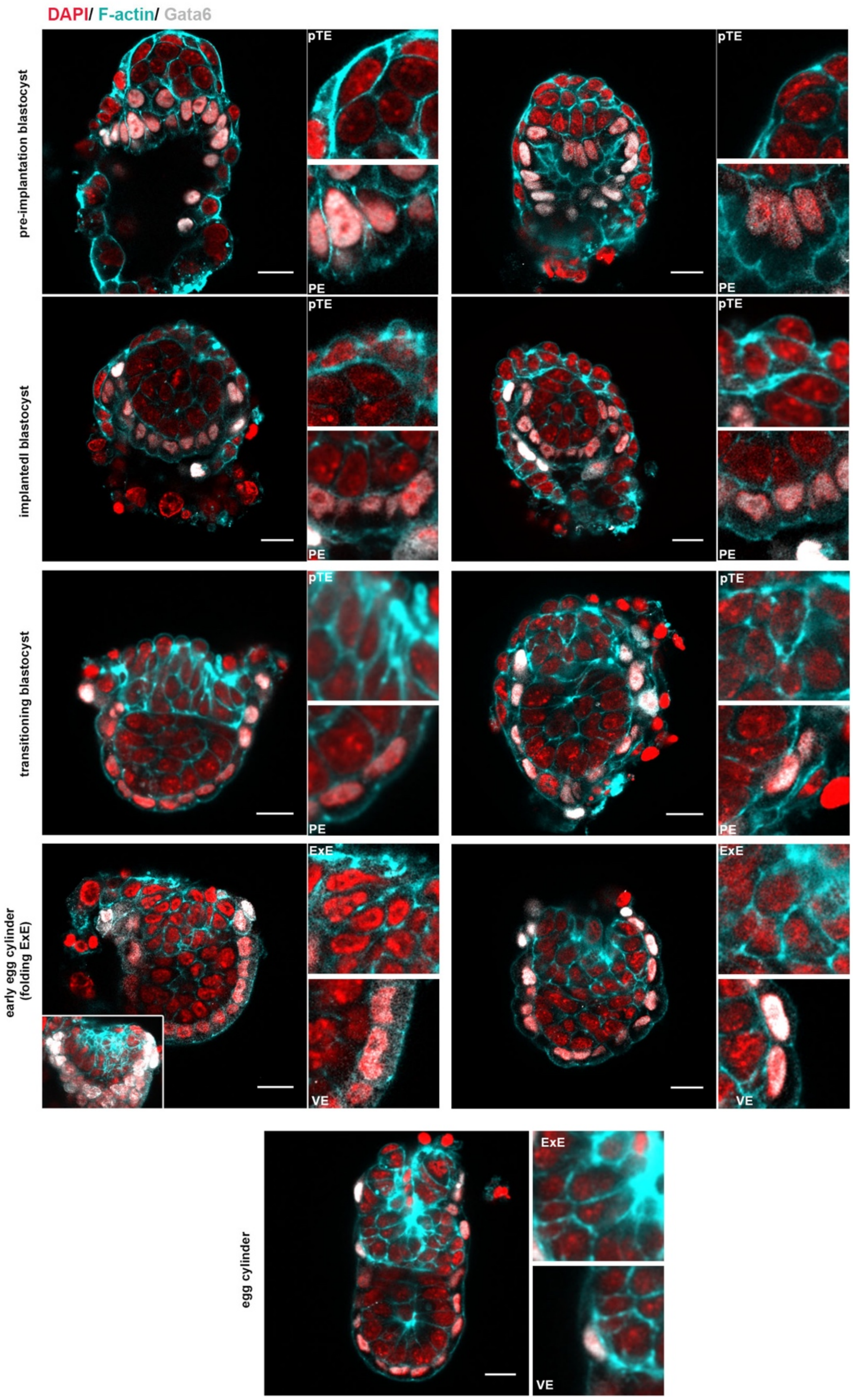

Supplementary Figure 8. Primitive endoderm cellular behaviour correlates with cell shape changes within the trophectoderm. Examples of embryos used for quantification of primitive endoderm (PE)/visceral endoderm (VE) and polar trophectoderm (TE)/extra-embryonic ectoderm (ExE) cell shape at different stages of development. Representative images of at least 20 embryos per stage. Scale bars=20um

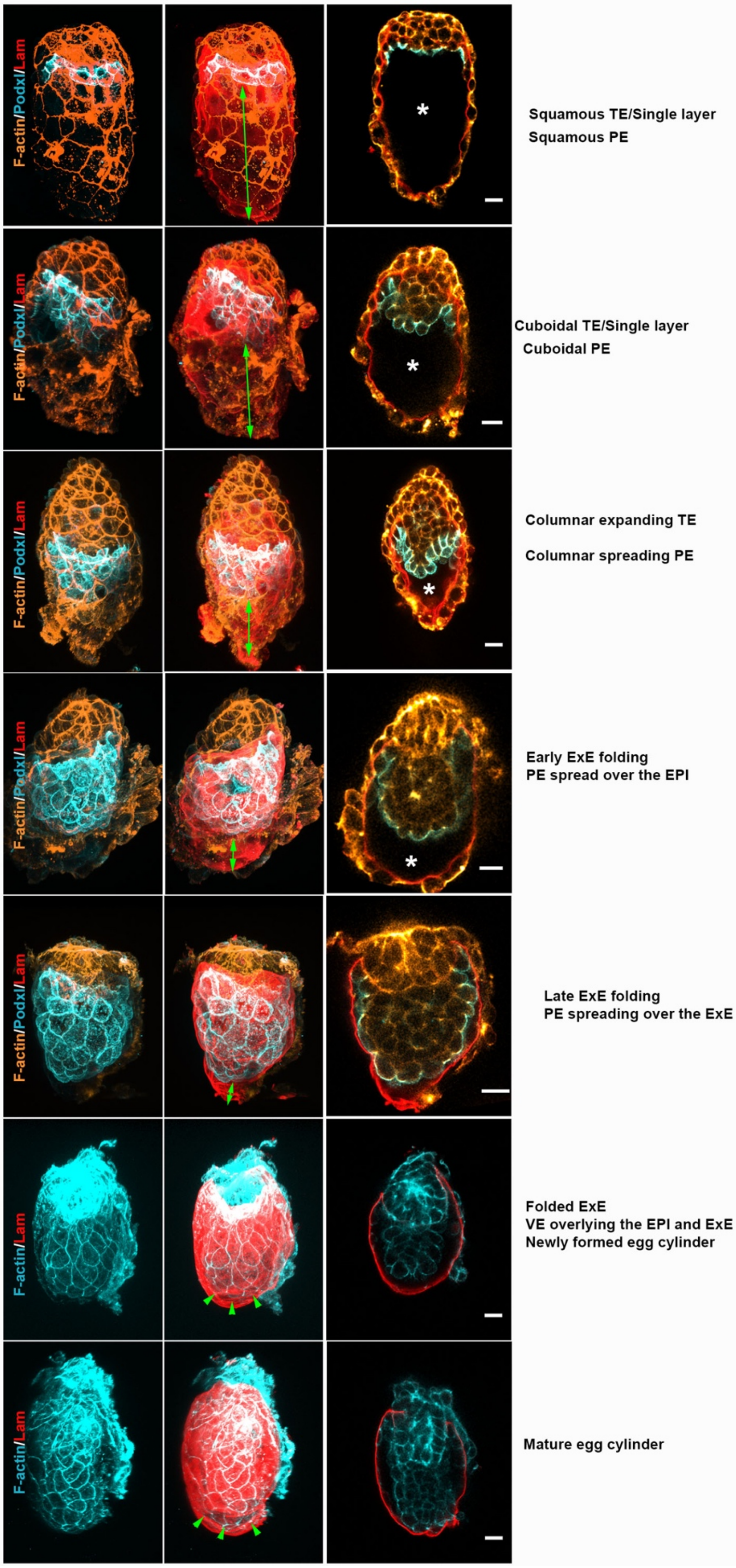
F-actin/Podxl/Lam
Squamous TE/Single layer
Squamous PE
F-actin/Podxl/Lam
Cuboidal TE/Single layer
Cuboidal PE
F-actin/Podxl/Lam
Columnar expanding TE
Columnar spreading PE
F-actin/Podxl/Lam
Early ExE folding
PE spread over the EPI
F-actin/Podxl/Lam
Late ExE folding
PE spreading over the ExE
F-actin/Lam
Folded ExE
VE overlying the EPI and ExE
Newly formed egg cylinder
F-actin/Lam
Mature egg cylinder

Supplementary Figure 9. Tissue positioning during the blastocyst to egg cylinder transition. Representative examples of embryos at different stages of peri-implantation development. First four rows: As the polar trophectoderm (TE) starts to expand forming the extraembryonic ectoderm (ExE), the epiblast and the visceral endoderm (VE)(cyan) are pushed within the blastocoel cavity (asterisk) towards the distal end of the Reichert's membrane (red). Last two rows: ExE folding results in further movement of the embryo towards the distal end of the Reichert's membrane (green arrowheads). The embryo occupies the whole blastocoel cavity upon egg cylinder formation. Double headed arrows: distance between PE and distal end of the Reichert's membrane at different developmental stages. Scale bar = 20um.

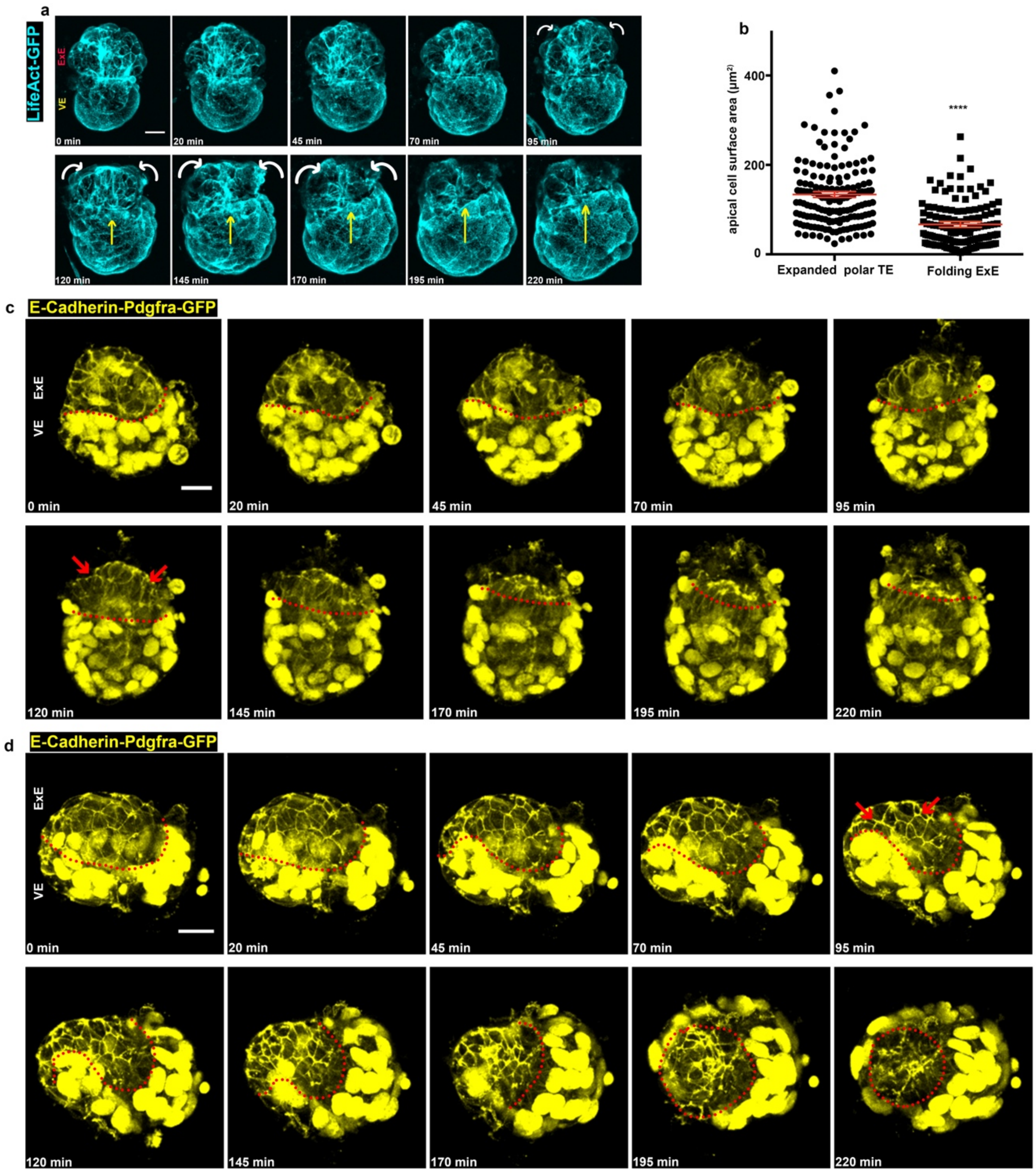

Supplementary Figure 10. Extra-embryonic ectoderm folding precedes egg cylinder formation. (a) Stills from a time lapse movie of a Lifeact-GFP E4.75 blastocyst. ExE tissue folding through apical constriction is followed by spreading of the visceral endoderm (VE) and formation of the egg cylinder. (b) Quantification of polar trophectoderm (TE) and extra-embryonic ectoderm (ExE) apical cell surface area before and during tissue folding. Two-sided unpaired student's t-test; ****P<0.0001; mean±SEM. Expanded polar TE: n=163 cells; Folding ExE: n=146**.** (c), (d) Stills from time lapse movies of Pdgfra/E-Cad GFP E4.9 blastocysts. Visceral endoderm (marked by Pdgfra) spreads to cover the newly formed extra-embryonic ectoderm only after extra-embryonic ectoderm folding through apical constriction. Dotted red line marks the proximal end of the visceral endoderm. Red arrowheads indicate the initiation of extra-embryonic ectoderm tissue folding. Source data are provided as a Source Data file. Scale bars=20um.

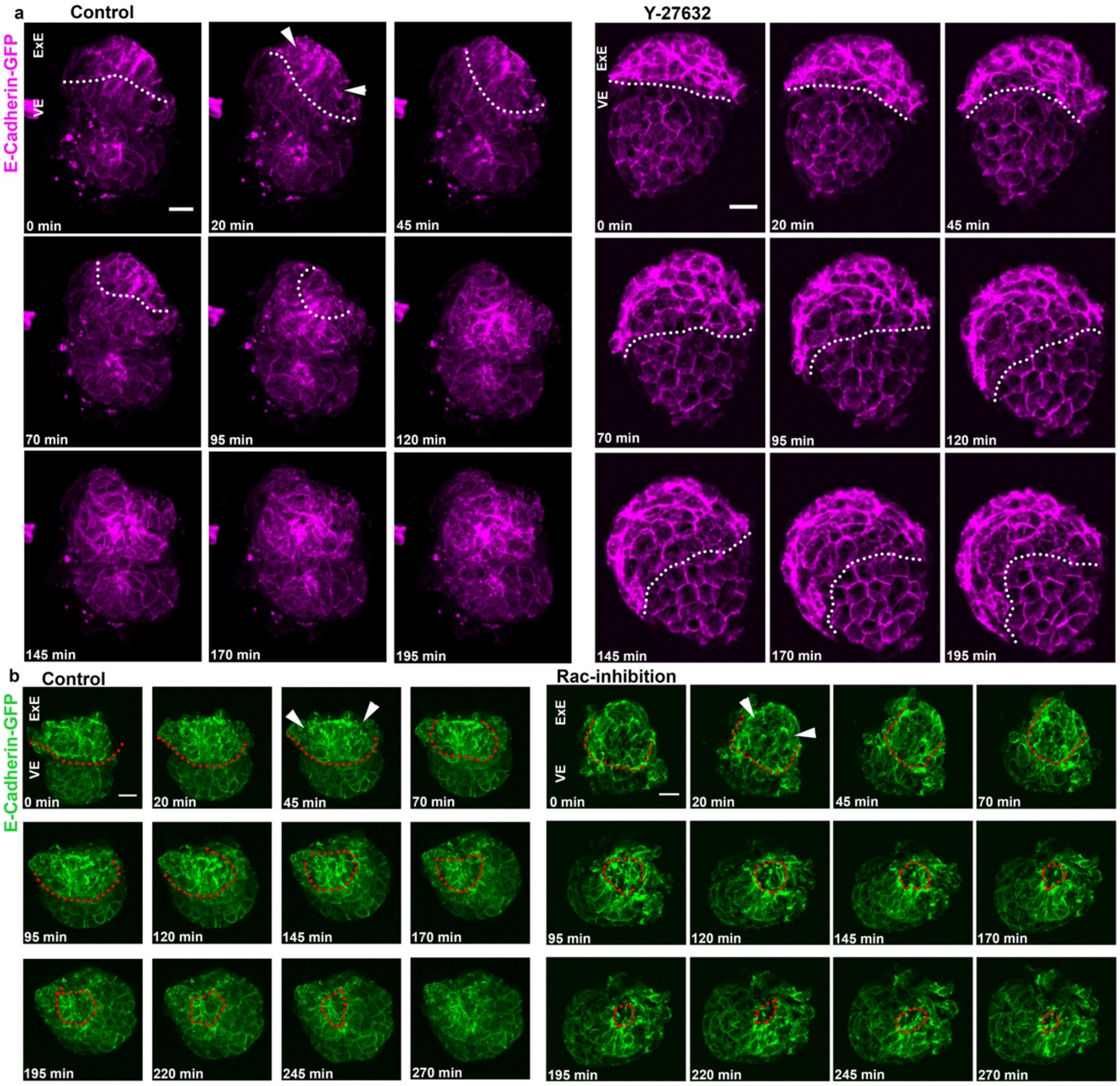

Supplementary Figure 11. Inhibition of extra-embryonic ectoderm folding results in defective egg cylinder formation. (a) Stills from time lapse movies of control and Y27632 (ROCK inhibitor) treated E-Cad GFP E4.9 blastocysts. ExE cells in ROCK inhibitor treated embryos fail to apically constrict and blastocyst to egg cylinder transformation fails. (b) Stills from time lapse movies of control and Rac inhibitor treated E-Cad GFP E4.9 blastocysts. Dotted lines in (a) and (b) marks the exposed extra-embryonic ectoderm region (not covered by the VE). Arrowheads in (a) and (b) mark initiation of ExE tissue folding. Scale bars= 20um

Supplementary methods

Live imaging of pre- and peri- implantation stage embryos

We adapted the in vitro culture protocol established by our lab[2], to optimise this system for blastocyst- and peri-implantation culture. Through this culture method, we have characterised the epiblast tissue morphogenesis from pre- to post-implantation[3]. Therefore, the entire method was optimised for epiblast growth. This includes removal of a part of the trophectoderm lineage to maximise culture fidelity[2].
To delineate the events driving the blastocyst to egg-cylinder transition, maintenance of both extraembryonic lineages is vital. To enable this, we developed a simple imaging technique for pre-implantation blastocysts, that allows imaging for up to 20h in 10 min time intervals, taking advantage of self-made imaging grids, that allow imaging of multiple positions, without the embryo moving out of position. To study the further development of the peri-implantation blastocyst into the post-implantation egg cylinder for up to 36h live imaging, we developed a simpler method based on thinly spread drops of medium on glass bottom dishes covered with mineral oil, that opens this developmental stage for analysis.

Reagents

Chemical	Supplier	Catalog number
M2 medium	Sigma Aldrich	M7167-100ML
KSOM	Sigma-Aldrich	MR-106-D
ADF	Thermo Fisher Scientific	12634-010
ITS-X	Thermo Fisher Scientific	51500-056
Glutamax	Life Technologies	35050-038
Penicillin-Streptomycin	Thermo Fisher Scientifics	15140122
Mineral oil	Biocare Europe SRL	9305
FBS	Thermo Fisher Scientific	10270-106
ddH2O		

Equipment

	Supplier	Catalog Number
1.5ml eppendorf	Fisher Scientific	0030120086
200ul pipet	Gilson Company, Inc.	F123601
200ul tips	Star Lab	S1120-8810-C
35x10mm petri dishes	SLS	351008
Bunsen burner/flame		
Confocal microscope	Leica SP8	
Dissection microscope	Nikon	

Forceps	Fine Science Tools	11254-20
Glass bottom, dish	MatTek Corporation	P35G-1.5-14-C
Pasteur Glass pipets	Thermo Fisher Scientific	11546963
Grids	Plastok	03-150/50
Heating stage	Agar Scientific	AG12857-220
Incubator Galaxy 170R	Eppendorf New Brunswick Scientific	CO17311022
Needle	Fisher Scientific	10703815
Pasteur pipets	VWR	612-1681
Scissors	Scientific Laboratie Supplies	ins4860
Silicone adhesive sealant		
Spinning disc	Zeiss	
Syringe	Thermo Fisher Scientific	10142104
Tubing for mouth-pipetting		

Supplementary References

1 Shahbazi, M. N. *et al.* Pluripotent state transitions coordinate morphogenesis in mouse and human embryos. *Nature* **552**, 239-243, (2017).

2 Bedzhov, I., Leung, C. Y., Bialecka, M. & Zernicka-Goetz, M. In vitro culture of mouseblastocysts beyond the implantation stages. Nat Protoc 9, 2732-2739, (2014).

3 Bedzhov, I. & Zernicka-Goetz, M. Self-organizing properties of mouse pluripotent cells initiate morphogenesis upon implantation. Cell 156, 1032-1044, (2014).
