# Supplementary material for: Morphogenesis of extra-embryonic tissues directs the remodelling of the mouse embryo at implantation
Source: Nat Commun. 2019 Aug 7;10:3557. doi: 10.1038/s41467-019-11482-5 (PMC6686005; doi:10.1038/s41467-019-11482-5)
Supplement: Supplementary file 3 — Description of Additional Supplementary Files [file 41467_2019_11482_MOESM3_ESM.docx]

**Description of Additional Supplementary Files**

**File Name: Supplementary Movie 1**

**Description:** *TE cell flow during pre-implantation development.* Time lapse movie of a representative E3.5 Cdx2-GFP transgenic embryo showing polar trophectoderm cell flow. Cyan dots indicate tracking of individual cells. Time interval=10 min; 121 time points.

**File Name: Supplementary Movie 2**

**Description:** *Trophectoderm cell flow stops upon embryo implantation*. Time lapse movie of a representative E4.5 Cdx2-GFP transgenic embryo showing that polar trophectoderm cell flow towards the mural trophectoderm stops upon implantation. Cyan dots: tracking of mural trophectoderm; Magenta dots: tracking of polar trophectoderm. Time interval=10 min; 38 time points.

**File Name: Supplementary Movie 3**

**Description:** *Trophectoderm cell flow stops upon embryo implantation*. Time lapse movie of a representative E4.5 Cdx2-GFP transgenic embryo showing that polar trophectoderm cell flow towards the mural trophectoderm stops upon implantation. Note the establishment of Cdx2 gradient in the trophectoderm. Time interval=10 min; 53 time points.

**File Name: Supplementary Movie 4**

**Description:** *Trophectoderm tissue boundary forms during embryo implantation.* Time lapse movie of a representative E4.5 Cdx2/E-cad GFP double transgenic embryo showing tissue boundary formation between polar and mural trophectoderm. Time interval=5 min; 40 time points.

**File Name: Supplementary Movie 5**

**Description:** *Polar trophectoderm expansion.* Time lapse movie of a representative E4.5 Lifeact-GFP transgenic embryo showing polar trophectoderm expansion following polar/mural trophectoderm tissue boundary formation. Time interval=5 min; 224 time points.

**File Name: Supplementary Movie 6**

**Description:** *Polar trophectoderm expansion*. Time lapse movie of a representative E4.5 Ecad-GFP transgenic embryo showing polar trophectoderm expansion; 173 time points.

**File Name: Supplementary Movie 7**

**Description:** *Cell shape changes within the primitive endoderm during implantation*. Time lapse movie of two representative E4.5 Lifeact-GFP transgenic embryos showing primitive endoderm cell behaviour before (left and right) and after (right) polar trophectoderm expansion. Time interval=5 min; 87 time points.

**File Name: Supplementary Movie 8**

**Description:** *Extraembryonic ectoderm folding is followed by visceral endoderm expansion.* Time lapse movie of three representative E4.75 Lifeact-GFP transgenic embryos showing extraembryonic ectoderm folding and visceral endoderm spreading during the last step of the blastocyst to egg cylinder transformation. Time interval=5 min; 57 time points.

**File Name: Supplementary Movie 9**

**Description:** *Extraembryonic ectoderm folding is followed by visceral endoderm expansion*. Time lapse movie of two representative E4.75 Pdgfra/Ecad-GFP double transgenic embryos showing extraembryonic ectoderm folding and visceral endoderm spreading during the last step of the blastocyst to egg cylinder transformation. Time interval=5 min; 97 time points.

**File Name: Supplementary Movie 10**

**Description:** *Inhibition of apical constriction results in defective egg cylinder formation.* Time lapse movie of representative control and ROCK inhibitor treated E4.75 Ecad-GFP transgenic embryos. Extraembryonic ectoderm folding and visceral endoderm spreading is defective in ROCK inhibitor treated embryos. Time interval=5 min; 60 time points.
